# A modified axillo-femoral perfusion for acute type a aortic dissection accompanied with lower limb malperfusion

**DOI:** 10.1186/s13019-020-1060-2

**Published:** 2020-01-09

**Authors:** Qianzhen Li, Liangwan Chen, Yue Shen, Jiahui Li, Yi Dong

**Affiliations:** Department of Cardiac Surgery, Union Hospital of Fujian Medical University, Fuzhou, Fujian China

**Keywords:** Aortic dissection, Lower limb malperfusion, Aortic surgery, Axillo-femoral perfusion

## Abstract

**Background:**

Lower limb malperfusion accompanied with acute type A dissection (AAD) is reported to be an independent predictor for mortality. Timely treatment is required. However, staged approach to restore the perfusion of the ischemic leg before aortic repair has a continuously increase risk of aortic rupture. Aortic repair under isolated axillary artery perfusion also has the risk of prolonging leg ischemia. Here we introduce our experience in performing axillo-femoral perfusion, which is supposed to bring benefits for treating lower limb malperfuison.

**Methods:**

Thirty patients who suffered AAD accompanied by lower limb ischemia enrolled in our study. All patients received aortic repair as soon as possible using the modified axillo-femoral perfusion approach. The cardiopulmonary bypass and cooling started with the right axillary artery perfusion. Then the femoral artery of the ischemic side was exposed and sewn to a graft connected with another inflow cannula. The rectal temperature was about 31 °C when the femoral perfusion started. The perfusion of the ischemic legs preoperative was estimated after the surgery by the clinical signs, the saturation of the distal-limb, and computed tomography scan.

**Results:**

Twenty-eight patients got good perfusion of the lower body after the surgery. Two patients received femoral-femoral artery bypass immediately after surgery because of the thrombosis in the right common iliac artery, without further injury. No peripheral vessels damage occurred, and no compartment fasciotomy or amputation needed. One patient died for the sepsis and the subsequent multi organ failure 28 days postoperative.

**Conclusions:**

The modified axllio-femoral perfusion could restore the lower limbs’ perfusion simultaneously during the aortic surgery without neither delaying dissection repair nor prolonging the ischemic time. It is a simple, but safe and effective technique.

## Background

Acute type A dissection (AAD) is a potential fatal aortic emergency, which results from a tear in the aortic wall intima that extends into the aortic wall media to create a false lumen and a dissection flap [1]. Malperfusion of visceral, renal, spinal, or iliofemoral arteries ischemia, is a common problem in AAD with an incidence of 30% [2]. Lower limb ischemia is reported to be about 15% [3], and is an independent predictor for mortality [4].

The resulting ischemia depends on the degree and duration of the obstruction as well as the presence of a collateral circulation. Timely treatment (< 6 h) is required, otherwise irreversible damage will occur, such as ostefacial compartment syndrome, or leg loss [5]. Therefore, it is very important to reduce the ischemic time. Usually, the proximal surgical repair will increase blood flow of the true lumen, and close the proximal tear entry the obstruction, which leads to the decompression of the false lumen. Thus, the lower limb’s ischemia could be relieved after the surgery. However, there are some other situations need to be considered. First, the ischemia would persist during the surgery. Second, the decompression of false lumen may be uncertain, especially when the distal aorta has another big tear entry. Third, the perfusion would not be restored in case the obstruction is caused by the thrombus, which is so-called static obstruction. In these cases, the surgical interventions (eg. thrombectomy plus graft implantation or graft bypassing) or percutaneous interventions (eg. balloon fenestration or stent implantation) are required after the aortic repair. And it will certainly prolong the ischemic time of the lower limbs. Therefore, some authors advocate staged approach with initial percutaneous or surgical intervention, to restore the perfusion of the ischemic leg as soon as possible, when the ischemic time is closed to 6 h or even more [6–9]. But this will delay the aortic repair, with a continuously increase risk of the aortic rupture [10], which was reported to be approximately 1% per hour in the first 24 h [11].

Therefore, there is not a routine treatment for the lower limb’s malperfusion. It should be more favorable if the malperfusion could be reserved during the aortic surgery. Here, we would like to introduce a modified axllio-femoral perfusion approach, which could restore the perfusion of the lower body as soon as possible, without delaying aortic repair. Moreover, it is more likely to resolve the artery occlusion permanently after the surgery. We believe this is a simple, but safe and practical technique.

## Patients and methods

### Patient population

From January 2010 to August 2018, 503 patients with acute type A aortic dissection (AAD) admitted to our center, 34 cases among them were accompanied by lower limb malperfusion. Fifteen patients (2 with lower limb ischemia) died for aortic rupture before sending to operation room, and 9 patients (2 with lower limb ischemia) refused surgery for personal reasons. The other 479 patients received open aortic repair, 30 cases among them suffered from lower limb ischemia. The aortic dissection involved from ascending aorta to common iliac artery. There were no chronic limb ischemia in these patients. The symptoms or clinical sings of lower extremity ischemia included paleness, pulselessness, cold distal skin, acrodynia, and cyanosis. Preoperative diagnosis was also based on echocardiography, computed tomography angiography (CTA), or magnetic resonance imaging. The preoperative characteristics are listed in Table 1.

**Table 1:** Preoperative characteristics

Characteristics	Value
Patients (*n*)	30
Age (y)	
Mean ± SD (range)	48.2 ± 6.5 (21–75)
Gender (*n*)	
Male / Female	25 / 5
Hypertension (*n*)	23
Diabetes (*n*)	4
Chronic renal dysfunction (*n*)	1
Cardiac tamponade (*n*)	5
Acute left heart failure (*n*)	1
Acute renal dysfunction (*n*)	4
Cerebral infarction (*n*)	1
Moderate or severe aortic valve regurgitation (*n*)	6
Mechanisms of the obstruction (*n*)	
True lumen compressed by the false lumen	24
Branch vessels obstructed by the intimal flap	4
Thrombosis	2
Interval time between the onset of lower limbs ischemia and operation (h)	
Mean ± SD (Range)	5.1 ± 1.9 (3.1–10.0)

### Surgical procedure

**Fig. 1:**
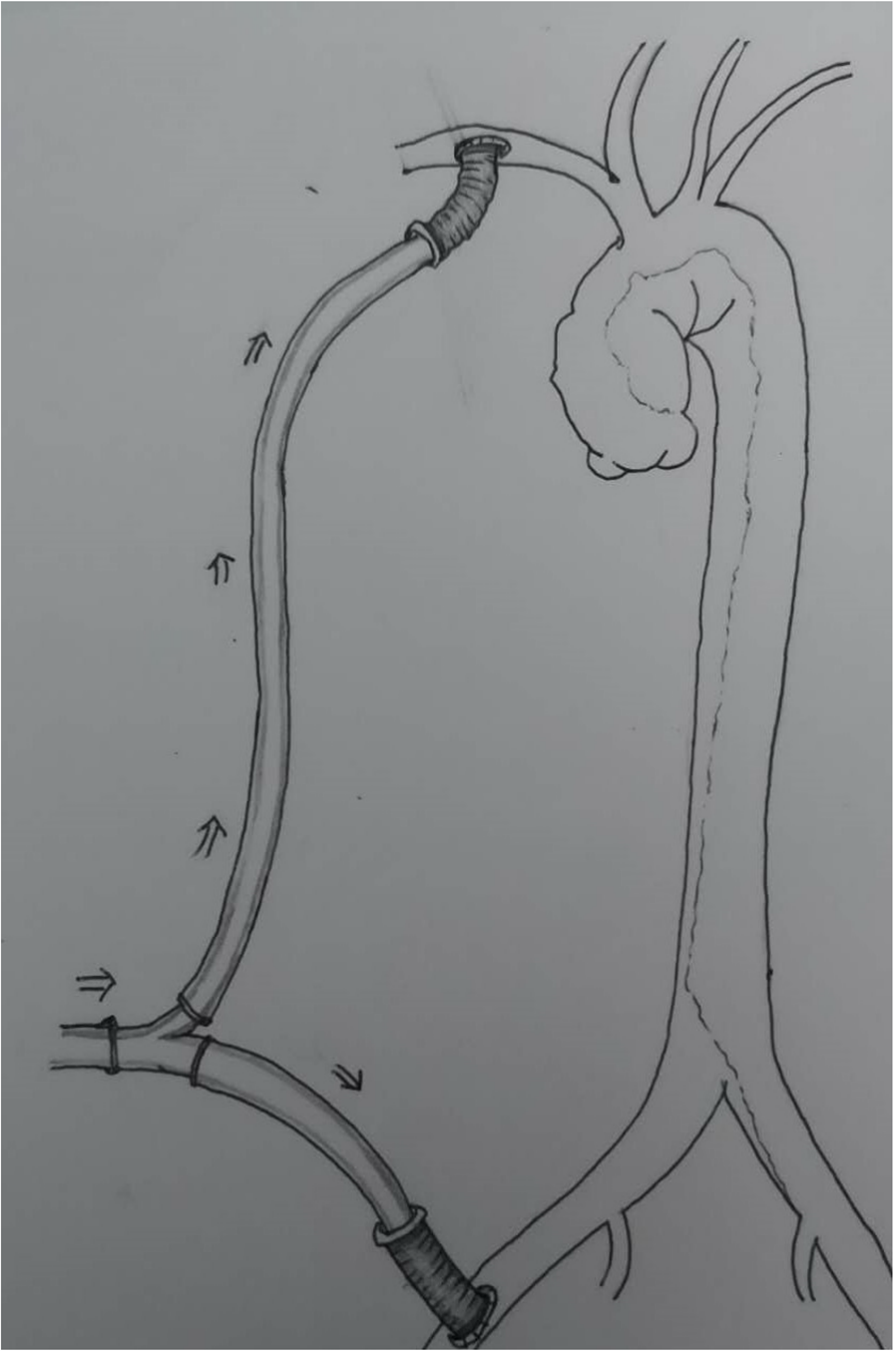
A short 8-mm Dacron polyester fabric graft was sewn to the right axillary and then connected to inflow cannula. After median sternotomy, cardiopulmonary bypass was established. During core cooling, the femoral artery of the ischemic site was exposed, and was sewn to another short 8-mm Dacron polyester fabric graft, followed with attaching to Y connector of right axillary artery cannulation

All patients were performed by the same surgeon. After the general anesthesia in the supine position, the blood pressure of both the upper and lower limbs was monitored, as well as the cerebral saturation, rectal temperatures. Near-infrared reflectance spectroscopy (NIRS) was also used to assess distal -limb perfusion of the ischemic limb [12]. The right axillary artery was exposed. After heparinization, a short 8-mm Dacron polyester fabric graft was sewn to the right axillary artery, and then connected to inflow cannula. Median sternotomy was performed, venous cannulation was set via superior and inferior vena cava or right atrium. Then cardiopulmonary bypass (CPB) and cooling was established. CPB flow was maintained between 2.4 and 2.6 L·min^− 1^·m^− 2^. During cooling, the femoral artery on the ischemic site was exposed, and was sewn to another short 8-mm Dacron polyester fabric graft, followed with attaching to Y connector of right axillary artery cannulation (Fig. 1). The rectal temperature of the onset of femoral perfusion was about 31 °C. Myocardial protection was achieved using cold blood cardioplegia. As we described previously [13–16], a proximal manipulation and replacement of ascending aorta was performed if necessary. When rectal temperature was cooled to a 22 °C, the lower body’s perfusion was discontinued, and selective cerebral perfusion via the right axillary artery was established at a rate of 10 to 15 mL·kg^− 1^·min^− 1^. Bilateral antegrade cerebral perfusion via the right axillary artery and left carotid artery could be applied in case poor cerebral lateral circulation was detected by cerebral CTA before the surgery.. The perfusion volume was controlled depending on the changes of the cerebral saturation. Then hemi-arch (2 cases) or total arch repair (28 cases) was performed. After CPB, protamine was used to reverse the heparinization, and the grafts were transected close to the anastomosis and the stumps were oversewn.

The postoperative perfusion of the lower limb was estimated by the clinic signs, NIRS, and CTs. Clinic signs of the good reperfusion included palpable pulses, redder or warmer of the distal skin. NIRS displayed saturation changes of the distal limb. CTs was performed to estimate the blood flow of the lower body.

## Results

The mean CPB time was 146.2 ± 36.5 (range, 110–200) minutes, aortic crossclamp time was 55.8 ± 8.8 (range, 45 to 119) minutes, and selective cerebral perfusion and lower body arrest time was 24.7 ± 6.9 (range, 15–28) minutes. The interval time between skin incision and femoral artery perfusion was 35.7 ± 3.5 (range 31–43) minutes. The interval time between the initiation of CPB and the femoral perfusion was 17.5 ± 3.3 (range 15–29) minutes.

**Fig. 2:**
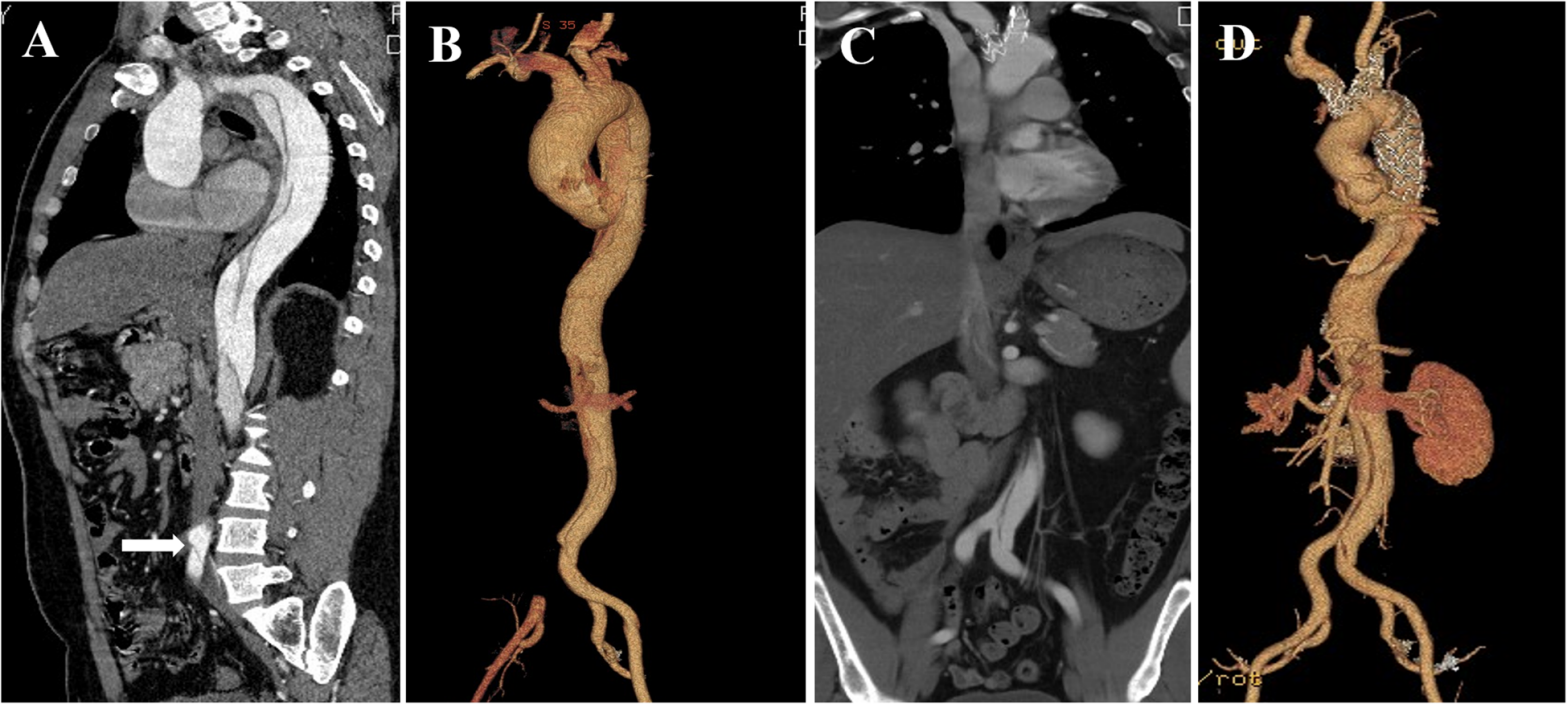
No pulse could be detected from the right lower limb of this patient before the surgery. The skin was colder and paler than another limb. CTs showed an antegrade propagation of the dissection from the ascending aorta to the left iliac artery, accompanied with a complete occlusion of the right common iliac artery, whose orifice was obstructed by a tense false lumen (arrow). Low-density enhanced of the right femoral artery was detected, probably due to the collateral circulation **a, b**. The patient was performed ascending aorta replacement and triple-branched stent graft implantation. Post-operative CT showed the perfusion of right common iliac artery was complete restored **c, d**

The postoperative data of the patients was list on the Table 2. The saturation of the distal-limb increased from 40.3 ± 3.2% (range 35–45%) to 57.3 ± 4.5% (range 49–68%). Twenty-seven patients got good perfusion of the lower body after the surgery. CTs images was shown in Fig. 2. Two patients received femoral-femoral artery bypass immediately after surgery because of the thrombus in the right common iliac artery, without further injury. No peripheral vessels damage occurred, and no compartment fasciotomy or amputation needed. One patient died for the sepsis and the subsequent multi organ failure 28 days postoperative.

**Table 2:** Postoperative data

Event	Value
Mean in-hospital time (days)	12.3 ± 4.4
Reoperation for hemostasis (*n*)	1
Stroke (*n*)	0
Gastrointestinal hemorrhage (*n*)	1
Acute renal failure/requiring hemodialysis (*n*)	4/2
Injury to the spinal cord (*n*)	0
Infection (*n*)	3
Lung	2
Sepsis	1
30-day post-operative mortality (*n/%*)	1/3.3

## Discussion

AAD can be complicated by visceral, renal, and lower limb ischemia, which are associated with higher mortality [2]. Patients with acute occlusion and a completely ischemic limb require intervention within six hours to prevent extensive myo-necrosis, neurologic injury and renal failure due to myoglobinuria. Securing circulation is the prime target for intervention [17].

There are several mechanisms for the malperfusion [18]. They can be classified as dynamic obstruction or static obstruction. Dynamic obstruction, such as the compression of true lumen by the false lumen, or the occlusion of a branch artery resulting in an intimal flap at the ostia of the vessel, or a circumferential tear with intimomedial intussusception, is the most commonly reason. Static obstruction, which is caused by the thrombosis, has less appearance.

Most of the time, the dynamic obstruction could be resolved by the decompression of false lumen and the increase flow of true lumen after repair of the proximal entry. However, the decompression of false lumen is uncertain if the obstruction is caused by thrombus or several big tear entries occurred in the distal aorta. It will certainly prolong the ischemic time of lower limbs, leading to irreversible damage to the ischemic legs. But initial intervention to restore the lower leg’s perfusion carries a substantial risk of aortic rupture before repair [10, 11]. Given this, some other surgeons suggest to make a femoro-femoral [19] or axillofemoral bypass [20, 21] simultaneously during the surgical repair. But this procedure requires one more incision in the other leg, which is still time consuming. Moreover, the graft increases thrombosis risk long-term after the surgery. Garret and Wolf [21] reported an axillo-bi-femoral graft to achieve reperfusion of the ischemic lower limbs caused by an occlusion of infrarenal aorta in 2006. From what they described, the proximal axillo-bi-femoral graft had to be sutured to the axillary arteriotomy after removing inflow cannulas. Different from these approaches, we just placed the cannula into the ischemic leg. And mostly, the flow of the ischemic leg would recover without no more secondary treatments or graft connection.

There are several accessible arteries for aortic perfusion, such as axilla artery, and femoral artery. Some surgeons highly recommend the axillary artery as the only inflow site during the aortic repair [22]. Generally, the flow is enough during the CPB. However, it is occasionally unreliable [23]. And the blood flow of lower body may be insufficient especially when there is a severe stenosis or occlusion in the distal aorta, which may delay the ischemic time. Others advocated the femoral artery as the only inflow site. But it can’t supply the perfusion of the upper body above the obstruction, and the potential emboli may drop and go upwards following the retrograde blood flow. These would probably increase the mortality and stroke rates [24] .

In our previous papers, we routinely used both the axillary artery and femoral artery as the inflow sites [13–16]. The dual-artery perfusion could provide good flow for the upper and lower body. And it should able to cool or rewarm the whole body more evenly. We modify this procedure. First, the inflow site of femoral artery must be the ischemic leg. Because the flow from the femoral cannulation could supply the perfusion of the lower body below the obstruction, without depending on the decompression of the false lumen. Moreover, the blood flow from femoral cannula would push aside the intima flap and increase the pressure of the true lumen immediately, which has highly possibility to avoid secondary treatment. Although the static obstruction required secondary intervention, it would not prolong the ischemic time of the lower limb during the surgery. Second, the axillary artery perfusion started before femoral artery perfusion may decrease the stroke incidence. Because the direct cold blood perfusion of lower body is supposed to reduce ischemia-reperfusion injury, according to the experiment studies which have shown that local hypothermia during early reperfusion protects skeletal muscle from ischemia-reperfusion injury [25]. According to above, this modified technique seems to get more benefits. The early outcoms were encouraged.

There are some limitations this technique. First, the true lumen of the femoral artery for the cannulation should be intact. Otherwise, the dissection may be extended, and the blood was not able to flow into the true lumen. Second, although the mean interval time between skin incision and femoral artery perfusion was less than 40 min, which was supposed to lead to very little ischemic injury, it is still time consuming. Surgeons should perform the procedure as quickly as possible. Furthermore, long-term follow up is required.

## Conclusions

In conclusion, for patients with AAD accompanied with lower limbs malperfusion, the modified axllio-femoral perfusion is a simple, but safe and effective method to simultaneously address the aortic dissection and the lower limb ischemia without neither delaying dissection repair nor prolonging malperfusion time. Moreover, it has the benefit of protective cold reperfusion of the ischemic leg, and a high probability to avoid secondary surgery in case the malperfusion is caused by dynamic obstruction.

## Abbreviations

AAD: Acute type A dissection
CPB: Cardiopulmonary bypass.
CTA: Computed tomography angiography.
NIRS: Near-infrared reflectance spectroscopy.
